# Drug-seeking motivation level in male rats determines offspring susceptibility or resistance to cocaine-seeking behaviour

**DOI:** 10.1038/ncomms15527

**Published:** 2017-05-30

**Authors:** Qiumin Le, Biao Yan, Xiangchen Yu, Yanqing Li, Haikun Song, Huiwen Zhu, Weiqing Hou, Dingailu Ma, Feizhen Wu, Yuqing Zhou, Lan Ma

**Affiliations:** 1 Department of Neurosurgery, and Institute of Translational Neuroscience, Huashan Hospital, State Key Laboratory of Medical Neurobiology, School of Basic Medical Sciences and Institutes of Brain Science, Fudan University, 138 Yixueyuan Road, Shanghai 200032, China; 2 Laboratory of Epigenetics, Institutes of Biomedical Sciences, Fudan University, 138 Yixueyuan Road, Shanghai 200032, China

## Abstract

Liability to develop drug addiction is heritable, but the precise contribution of non-Mendelian factors is not well understood. Here we separate male rats into addiction-like and non-addiction-like groups, based on their incentive motivation to seek cocaine. We find that the high incentive responding of the F0 generation could be transmitted to F1 and F2 generations. Moreover, the inheritance of high incentive response to cocaine is contingent on high motivation, as it is elicited by voluntary cocaine administration, but not high intake of cocaine itself. We also find DNA methylation differences between sperm of addiction-like and non-addiction-like groups that were maintained from F0 to F1, providing an epigenetic link to transcriptomic changes of addiction-related signalling pathways in the nucleus accumbens of offspring. Our data suggest that highly motivated drug seeking experience may increase vulnerability and/or reduce resistance to drug addiction in descendants.

## Abstract (web summary)

Drug addiction is partially heritable but the non-genetic inheritance mechanisms are not well understood. The authors show that motivation of male rats in response to cocaine self-administration elicit susceptibility and/or decreased resistance to developing addiction like behaviour in offspring.

Acquisition of nutritional resources is one of the critical driving forces in evolution^1^. Pursuit of food and water, which produces positive effects on the reward system, are reinforced and inherited as natural rewards. Dynamic interactions between genes and experiences are involved in the selective adaptation to new potential rewarding stimuli during evolution^2^. Emerging evidence indicates that, in addition to natural selection of genetic variants, non-Mendelian epigenetic changes induced by ancestral environmental and behavioural perturbations also contribute to the transgenerational inheritance of adaptive phenotypes^3^,^4^,^5^,^6^.

Addictive substances of abuse, such as cocaine and heroin, induce much stronger rewarding effects than natural rewards. Chronic consumption of such drugs can lead to addiction, which is characterized by an increase in consumption of drugs with enhanced motivation at the expense of individual's health and social life^7^,^8^. The major substrates that cause persistent compulsive drug use are attributed to molecular and cellular adaptations that occur in the mesolimbic system, which largely overlaps with the nature reward circuit^9^.

Indeed, epidemiological studies show a remarkable degree of familial aggregation of substance abuse^10^,^11^. Large-scale twin and adoption studies have demonstrated that the heritability of liability for substance abuse is between 50 and 60% (refs 12, 13, 14, 15). The high heritability has been largely attributed to, but not fully explained by, variations in the genome^16^. We thus hypothesized that being addicted to drug(s) of abuse may promote epigenetic adaptation in favour of a positive responding (for example, enhanced motivation to seek drug reinforcement) to the drug reward, resulting in an increased vulnerability to addiction in subsequent generations.

Epidemiological studies show that <20% of drug users become addicted^14^,^17^, which was confirmed by laboratory animal experiments^18^. Here in an attempt to explore the effect of cocaine-induced reinforcement in subsequent generations, we divide F0 rats into two groups by their incentive responding to cocaine reward. We show that F1 and F2 offspring sired by rats with prior experience of high motivational self-administration of cocaine (which we term Addict rats) exhibit increased susceptibility or reduced resistance to cocaine-seeking behaviour, and that this transgenerational effect is dependent on high incentive motivation induced by cocaine, but not high intake of cocaine *per se*. Reduced representation bisulfite sequencing reveal persistent changes in DNA methylation of sperm DNA in F0 and F1 of rats displaying addiction-like behaviour, suggesting possible involvement of epigenetic mechanisms in this behavioural effect.

## Results

### Cocaine reinforcement is transgenerationally inherited

**Figure 1:**
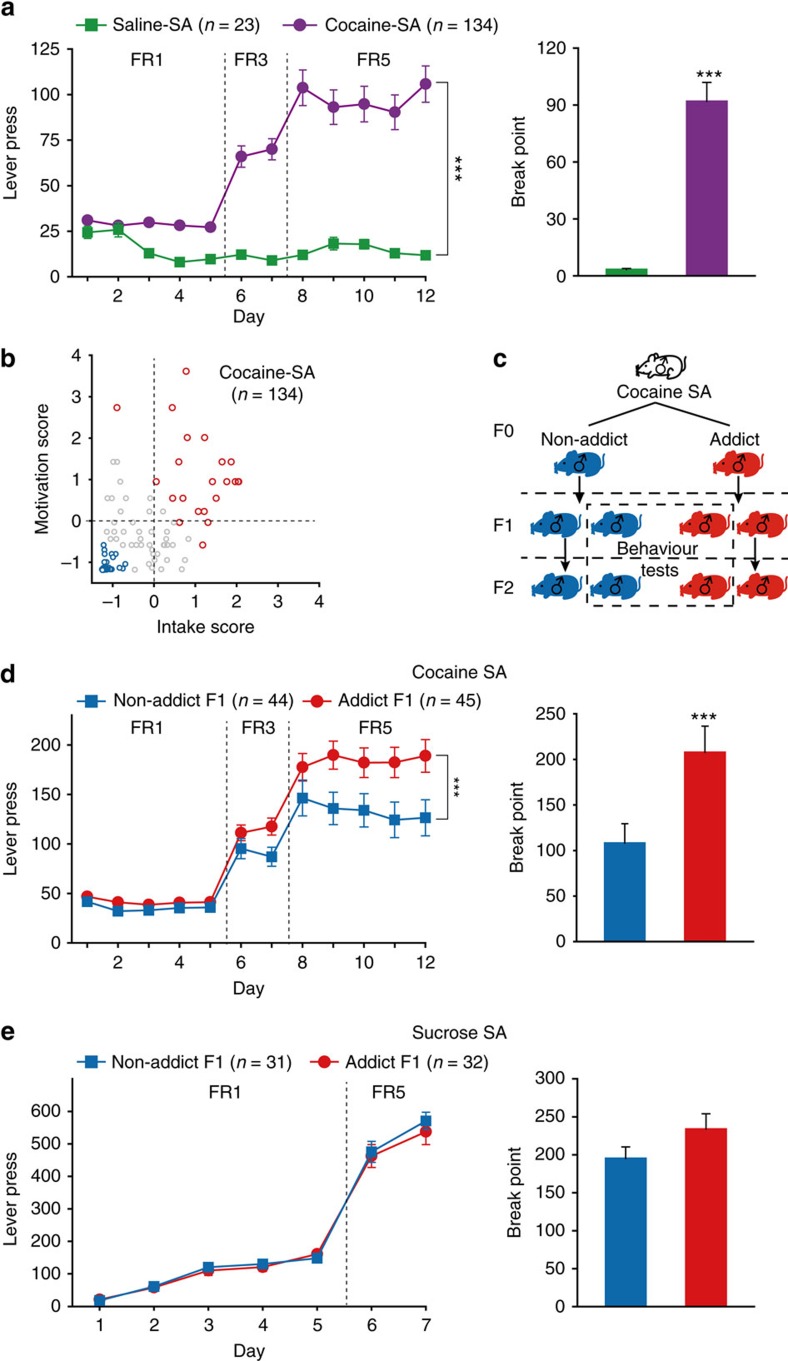
Performance of Addict and Non-addict F0 rats in cocaine self-administration. (**a**) Naive rats were trained to self-administer saline (Saline SA) or cocaine (Cocaine SA) under a 5-day FR1, 2-day FR3, 5-day FR5, 1-day PR schedule. Cocaine-SA group showed higher lever presses at FR (left) and break point (right) at PR schedules, compared with Saline-SA group. Lever press, Group × FR, mixed model repeated measures (MMRM), *χ*^2^ (2)=101.91, *P*<0.001; Group, ***P<0.001, Saline-SA versus Cocaine-SA. Break point, Mann–Whitney Rank-Sum Test, *Z*=4.688, ***P<0.001. Saline-SA, *n*=23; Cocaine-SA, *n*=134. (**b**) Scores for cocaine intake and motivation of each Cocaine-SA rat were calculated according to SA performance at FR5 and PR schedules, respectively, and plotted. Cocaine SA rats with a combined drug-seeking behavioural score within the top 25% and the bottom 40% were designated as Addict F0 (red circle) and Non-addict F0 (blue circle), respectively. (**c**) Addict F0 and Non-addict F0 rats were crossed with naive female rats to generate Addict F1 and Non-addict F1, which were then randomly designated for mating to generate F2 or for behavioural testing. (**d**) Addict F1 rats displayed higher cocaine consumption and motivation than Non-addict F1. Lever press, Group × FR, MMRM, *χ*^2^ (2)=23.33, *P*<0.001; Group, *P*=0.041; FR5, ***P<0.001, Non-addict F1 versus Addict F1. Break point, Mann–Whitney Rank-Sum Test, *Z*=3.272, ***P<0.001. Non-addict F1, *n*=44; Addict F1, *n*=45. (**e**) There was no difference in intake and motivation for sucrose reward in F1. Lever press, Group × FR, MMRM, *χ*^2^ (1)=10.1, *P*=0.32. Break point, Mann–Whitney Rank-Sum Test, *Z*=1.772, *P*=0.076. Non-addict F1, *n*=31; Addict F1, *n*=32. Data are expressed as mean±s.e.m.

To explore the potential transgenerational effects of cocaine-induced reinforcement, naive male Sprague-Dawley rats were allowed to self-administer cocaine (0.75 mg kg^−1^ per injection, Cocaine-SA group) or saline (Saline-SA group) by pressing the active lever in 4-h daily sessions. Rats were first subjected to self-administration at a fixed ratio (FR) of 1 (that is, one lever press for an injection), then switched to FR3 and FR5 (three and five lever presses for one injection, respectively) on days 6 and 8, and finally tested on a progressive ratio (PR) schedule on day 13 to assess their motivation for drug reinforcement. Compared with Saline-SA group, Cocaine-SA rats showed significantly higher lever presses during the FR schedules (Fig. 1a left) and higher break point (the number of lever presses to earn the last cocaine injection) in the PR test (Fig. 1a right).

The individual performance of each of the 134 Cocaine-SA rats in cocaine self-administration was analysed. Uncontrollable urges to seek drugs and excessive drug consumption are important characteristics of addiction. Thus, to reflect the incentive motivational responding to cocaine reward, a combined behavioural score was calculated for each Cocaine-SA rat, by addition of normalized score derived from average lever pressing during FR5 program, and break point during PR schedule. Cocaine-SA rats with the top 25% and the bottom 40% of the behavioural score were designated as Addict F0 (red circle) and Non-addict F0 (blue circle), respectively (Fig. 1b).

**Figure 2:**
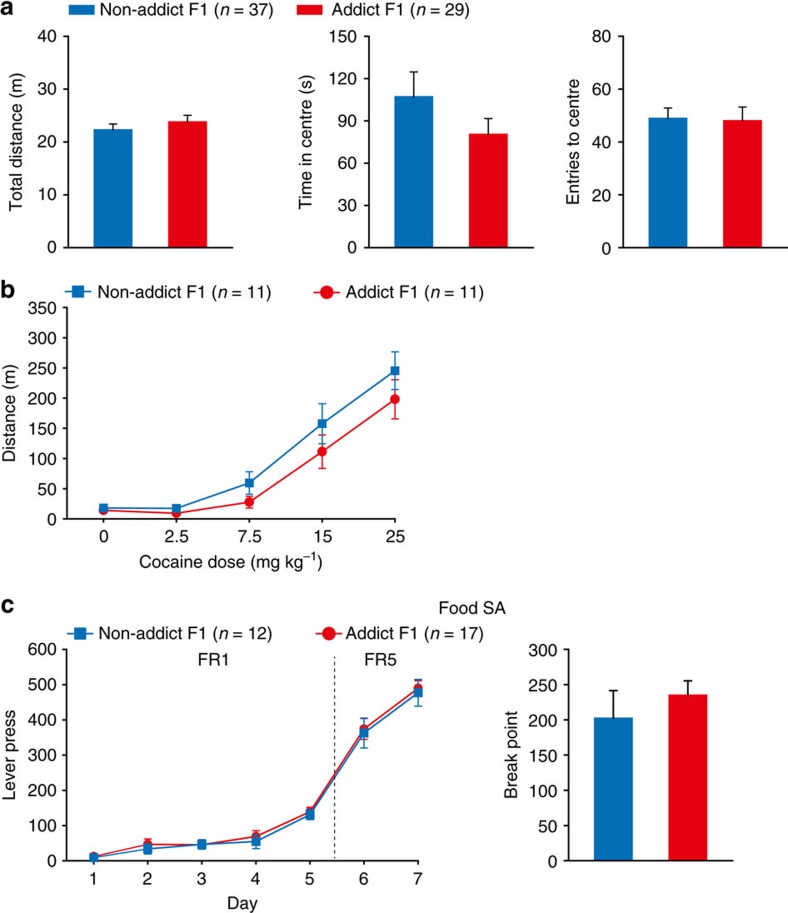
Comparable performance of Addict F1 and Non-addict F1 rats in basal and cocaine-induced locomotion activity and food self-administration. (**a**) Open field test. Total distance, Student's *t*-test, *t* (64)=0.872, *P*=0.386. Time in centre, Student's *t*-test, *t* (64)=1.197, *P*=0.236. Entries to centre, Student's *t*-test, *t* (64)=0.142, *P*=0.887. Non-addict F1, n=37; Addict F1, *n*=29. (**b**) Sensitivity to cocaine-induced locomotor activity. Two-way RM ANOVA, *F*_Group × Dose_ (4, 80)=0.734, *P*=0.571. Non-addict F1, *n*=11; Addict F1, *n*=11. (**c**) Food self-administration test. Lever press, Group × FR, MMRM, *χ*^2^ (1)=0.19, *P*=0.663. Break point, Mann–Whitney Rank-Sum Test, *Z*=1.039, *P*=0.299. Non-addict F1, *n*=12; Addict F1, *n*=17. Data are expressed as mean±s.e.m.

The Addict and Non-addict (F0) rats were then mated with naive female rats (Fig. 1c). Addict and Non-addict F1 rats (17 and 16 litters respectively) were obtained, and 2–6 adult male rats from each litter were subjected to the cocaine self-administration test. The F1 offspring of Addict rats, as compared with Non-addict F1, exhibited higher lever presses for cocaine injections during the FR program, and higher motivation for cocaine reinforcement as indicated by break point (Fig. 1d). Interestingly, the third litter of Addict F0, conceived after 6–7 weeks of abstinence, still showed higher voluntary cocaine intake in FR5 sessions compared with the third litter from Non-addict rats (Supplementary Fig. 1). Hyperactivity, reduced cocaine sensitivity and enhanced reward learning ability may increase lever presses in self-administration. Therefore, we conducted three series of experiments to exclude the possibility that any of the above-mentioned factors might have influenced our behavioural readout. In the open field test, no difference in basal locomotor activity and anxiety level between Addict and Non-addict F1 rats was observed (Fig. 2a). Moreover, Addict F1 and Non-addict F1 showed similar dose-dependent responses to cocaine in the sensitivity test (Fig. 2b). Finally, in sucrose and food (natural rewards) self-administration tests, the two groups exhibited equivalent self-administration learning curves and break points (Figs 1e and 2c).

**Figure 3:**
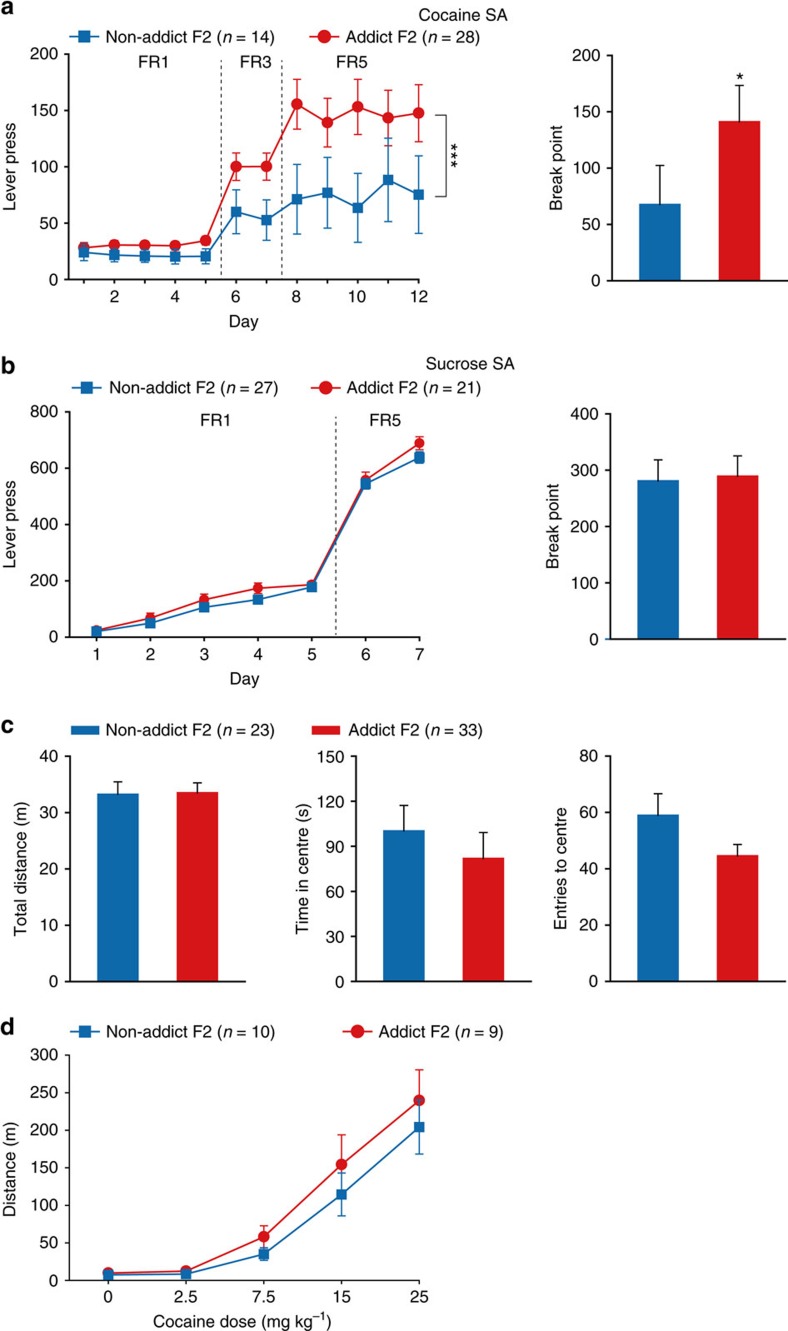
Addict F2 displayed higher responding to cocaine-induced reinforcement than Non-addict F2. (**a**) Addict F2 rats displayed higher cocaine consumption and motivation than Non-addict F2. Lever press, Group × FR, MMRM, *χ*^2^ (2)=28.63, *P*<0.001; Group, *P*=0.052; FR5, ***P<0.001, Non-addict F2 versus Addict F2. Break point, Mann–Whitney Rank-Sum Test, *Z*=2.513, *P=0.013. Non-addict F2, *n*=14; Addict F2, n=28. (**b**) Sucrose self-administration. Lever press, Group × FR, MMRM, *χ*^2^ (1)=0.29, *P*=0.588. Break point, Mann–Whitney Rank-Sum Test, *Z*=0.783, *P*=0.433. Non-addict F2, *n*=27; Addict F2, *n*=21. (**c**) Open field test. Total distance, Student's *t*-test, *t* (54)=0.0964, *P*=0.924. Time in centre, Student's *t-*test, *t* (54)=0.729, *P*=0.469. Entries to centre, Student's *t-*test, *t* (54) =−1.796, *P*=0.0781. Non-addict F2, *n*=23; Addict F2, *n*=33. (**d**) Cocaine-induced locomotor activity. Two-way RM ANOVA, *F*_group × day_ (4, 68)=1.170, *P*=0.295. Non-addict F2, *n*=10; Addict F2, *n*=9. Data are expressed as mean±s.e.m.

The phenotype observed in F1 generation could arise from direct exposure of the sperm of F0 rats to cocaine rather than from transgenerational transmission mechanisms. To exclude this possibility, Addict F2 and Non-addict F2 rats, sired by naive F1, were subjected to evaluations in cocaine self-administration. Similar to our observations in their F0 and F1 ascendants, Addict F2 rats displayed higher lever presses under FR5 schedule, and higher break point than Non-addict F2 (Fig. 3a), while their performance in sucrose self-administration (Fig. 3b), open field (Fig. 3c), and their response to acute cocaine doses was comparable to Non-addict F2 group (Fig. 3d). These data suggest that paternal propensity to develop high cocaine-seeking behaviour could be transgenerationally maintained in subsequent generations.

### Motivation determines cocaine response in offspring

**Figure 4:**
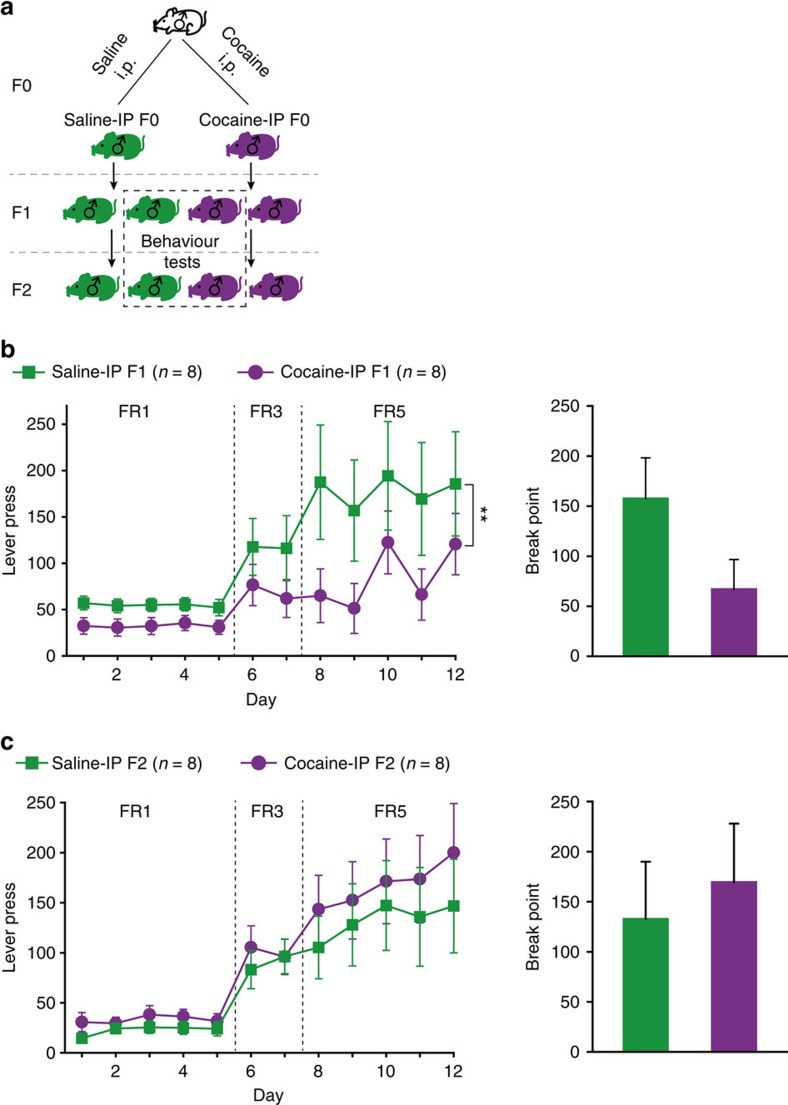
Non-contingent chronic cocaine injections induce inter-generational cocaine resistance. (**a**) Rats received 15 mg kg^−1^ cocaine (Cocaine-IP F0) or equal volume of saline (Saline-IP F0) intraperitoneal injections twice daily for 7 days and were mated with naive females one day after the last injection. Four litters from each of group were randomly designated for behavioural experiment or for mating to generate F2. (**b**) Cocaine-IP F1 did not show an increase but displayed a decrease tendency in lever press and break point for cocaine in self-administration test, compared with Saline-IP F1 rats. Lever press, Group × FR, MMRM, *χ*^2^ (2)=13.00, *P*=0.0015; FR5, **P=0.009, Saline-IP F1 versus Cocaine-IP F1. Break point, Mann–Whitney Rank-Sum Test, *Z*=1.060, *P*=0.289. Saline-IP F1, *n*=8; Cocaine-IP F1, *n*=8. (**c**) There was no statistical difference in lever press or break point between Saline-IP F2 and Cocaine-IP F2. Lever press, Group × FR, MMRM, *χ*^2^ (2)=2.60, *P*=0.272. Break point, Mann–Whitney Rank-Sum Test, *Z*=0.638, *P*=0.526. Saline-IP F2, *n*=8; Cocaine-IP F2, *n*=8.

An alternative interpretation for the transgenerational effect is that Addict F0 self-administered more cocaine than Non-addict F0. Thus, inheritance could stem from the exposure to cocaine per se and may not represent incentive motivational effect of cocaine. To examine the effect of cocaine exposure, another cohort of naive rats was given twice-daily intraperitoneal injections of cocaine (15 mg kg^−1^, Cocaine-IP) or saline (Saline-IP) for 7 days and the response of their F1, F2 offspring in cocaine self-administration was tested (Fig. 4a). Interestingly, Cocaine-IP F1 sired by rats receiving non-contingent cocaine injections showed significantly lower responses than Saline-IP F1 rats (Fig. 4b), while the F2 offspring of Cocaine-IP and Saline-IP exhibited no difference in cocaine self-administration (Fig. 4c). Moreover, as shown in Supplementary Fig. 2, cocaine response of Addict F1 group was significantly higher than Saline F1 under FR5 schedule, and Non-addict F1 exhibited lower breakpoint than Saline F1. These data suggest that chronic cocaine intake results in lower drug responding in F1 generation, that is, a protective effect, and that the high response for cocaine-induced reinforcement observed in Addict offspring is unlikely caused solely by excessive exposure to high doses of cocaine. Previous studies have shown that only a portion of drug users or animals subjected to self-administration of addictive drugs become addicted^17^,^18^. Combined with the result of Vassoler *et al*.^19^ showing that F1 offspring of rats that self-administered cocaine exhibit lower responding than F1 from saline exposed animals, we speculate that the transgenerational transmission of high cocaine incentive response observed may be dependent on the addiction-like state in the F0 generation, and the incentive motivational effect causes increased susceptibility to cocaine-seeking behaviour or decreased protective effect in offspring.

**Figure 5:**
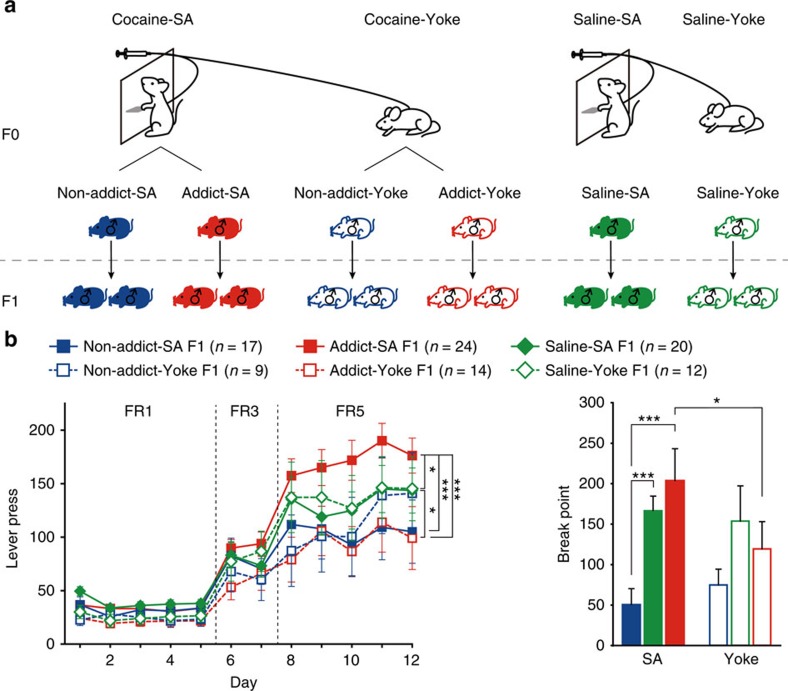
The inheritance of vulnerability to cocaine reinforcement is contingent on highly motivated cocaine self-administration. (**a**) Naive male rats were randomly paired. In each pair, one rat was allowed to actively self-administer cocaine or saline by pressing the lever (SA group), and with each drug infusion by SA group rat, a yoked infusion of exactly the same dose of cocaine or saline (Saline-Yoke) was delivered at the same time passively (without lever press) to the paired rat (Yoke group). The yoked rats corresponding to Saline-SA, Addict-SA and Non-addict-SA groups were designated as Saline-Yoke, Addict-Yoke and Non-addict-Yoke, respectively. (**b**) Addict-SA F1 rats showed significantly higher lever press and break point for cocaine than Non-addict-SA F1, but there was no difference between Addict-Yoke F1 and Non-addict-Yoke F1. Lever press, Group × Yoke × FR, MMRM, *χ*^2^ (4)=35.55, *P*<0.001; *P*<0.001, Saline F1 versus Non-addict F1, *P*<0.001, Non-addict F1 versus Addict F1; FR5, ***P<0.001, Addict-SA F1 versus Non-addict-SA F1, *P=0.013, Addict-SA F1 versus Saline-SA F1, ***P<0.001, Addict-SA F1 versus Addict-Yoke F1; *P=0.038, Addict-Yoke F1 versus Saline-Yoke F1. Break point, two-way ANOVA by Ranks, *F*_Group × Yoke_ (2, 91)=3.604, *P*=0.090; ***P<0.001, Addict-SA F1 versus Non-addict-SA F1, ***P<0.001, Non-addict-SA F1 versus Saline-SA F1, *P=0.041, Addict-SA F1 versus Addict-Yoke F1. Non-addict-SA F1, *n*=17; Non-addict-Yoke F1, *n*=9; Addict-SA F1, *n*=24; Addict-Yoke F1, *n*=14; Saline-SA F1, *n*=20; Saline-Yoke F1, *n*=12. Data are expressed as mean±s.e.m.

To further discriminate the effects of non-contingent and motivational intake of cocaine, yoked cocaine infusion experiment was carried out. In yoked cocaine delivery procedure, one rat self-administered cocaine by actively pressing the lever (SA), while the paired rat (Yoke) passively received the same dose of cocaine at the same time (Fig. 5a). Addict and Non-addict F0 in SA, as determined by the combined score, and their paired Yoke groups, were mated to generate F1. In contrast to the significantly higher drug intake and motivation for drug reinforcement observed in F1 of Addict-SA group compared with Non-addict-SA group, no difference in lever presses or break point for cocaine was observed between Addict-Yoke F1 and Non-addict-Yoke F1 rats (Fig. 5b). Non-addict-SA F1, but not Addict-SA F1, exhibited lower FR5 responding and break point than Saline-SA F1 rats (Fig. 5b), suggesting a loss of a protective effect in the Addict-SA F1 group. Each pair of Addict-SA and Addict-Yoke F0 rats received the same amount of cocaine simultaneously; however, the offspring of the two groups exhibited significant difference in lever press for cocaine. There was no difference in cocaine self-administration found between offspring of Saline-SA and Saline-Yoke groups. These results support the notion that high incentive motivation for cocaine, but not the intake of an addictive drug itself, renders transgenerational inheritance of vulnerability and/or reduce resistance to drug addiction in descendants.

**Figure 6:**
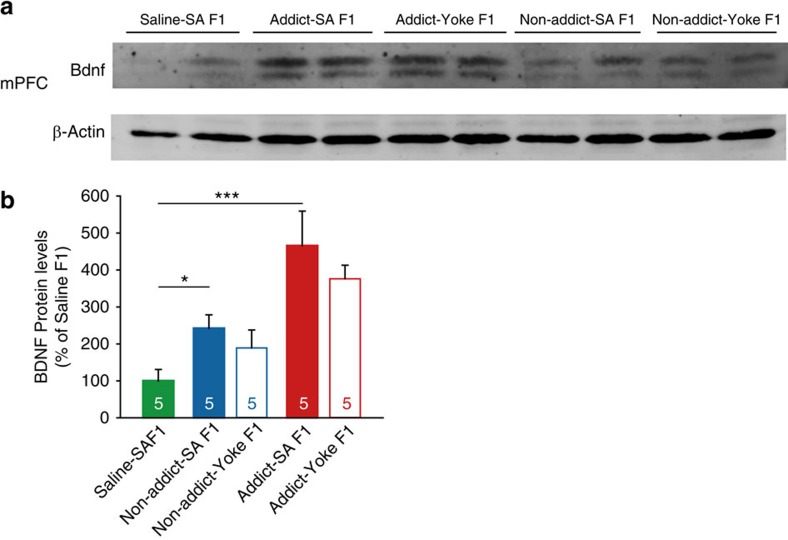
Elevated BDNF level in F1 correlates with the amount of cocaine taken by F0 fathers. BDNF level in mPFC of cocaine naive F1 rats of Saline-SA, Non-addict-SA, Non-addict-Yoke, Addict-SA and Addict-Yoke groups. (**a**) Representative blotting of two samples from each group was shown. (**b**) Statistical analysis of BDNF level in each group. BDNF level normalized by β-actin was acquired, and then relative protein level of each sample was divided by average of BDNF/β-actin in Saline F1 group. One-way ANOVA, *F* (4, 20)=7.251, *P*<0.001; Bonferroni *post hoc*, ***P<0.001, compared with Saline F1. *n*=5 each. Data are expressed as mean±s.e.m.

As Vassoler *et al*.^19^ found that the cocaine-resistant phenotype was attributed to increase in BDNF level of F1 male cocaine sired rats, we also measured BDNF level in mPFC of drug- and experiment- naive F1 rats from Saline, Non-addict-SA, Non-addict-Yoke, Addict-SA and Addict-Yoke groups (Fig. 6). The results showed that, F1 descendants of cocaine-experienced fathers (Non-addict-SA, Non-addict-Yoke, Addict-SA, and Addict-Yoke rats) showed cocaine dose-dependent elevation of BDNF level, compared with F1 offspring of Saline-SA group. However, BDNF level between SA and yoked rats from Addict or Non-addict groups was not different. These data indicate that paternal cocaine experience induces BDNF elevation in F1 offspring, and this is correlated with the amount of cocaine taken by F0 fathers, not motivation for reinforcement.

### Inheritance of drug seeking behaviour is an acquired trait

**Figure 7:**
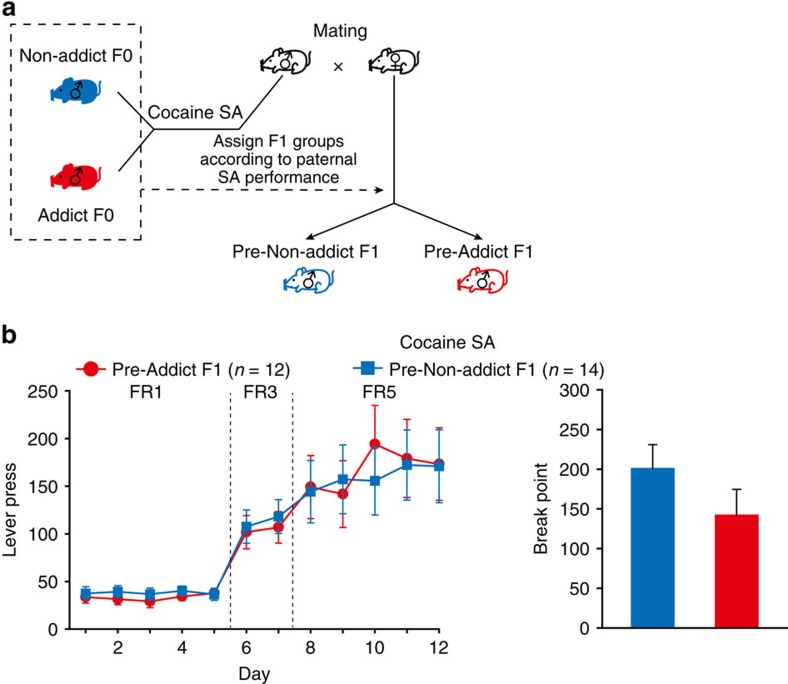
Transgenerational inheritance of cocaine reinforcement is an acquired trait. (**a**) Naive male rats were crossed with naive females first, and one week after mating were subjected to cocaine self-administration procedures to identify Addict and Non-addict rats according to their cocaine intake and motivation scores. The offspring sired by Addict and Non-addict rats (before their exposure to cocaine) were designated as Pre-Addict F1 and Pre- Non-addict F1. (**b**) Pre- Non-addict F1 and Pre-Addict F1 rats showed no difference in lever press and break point. Lever press, Group × FR, MMRM, *χ*^2^ (2)=0.79, *P*=0.68. Break point, Mann–Whitney Rank-Sum Test, *Z*=1.273, *P*=0.203. Pre- Non-addict F1, *n*=14; Pre-Addict F1, *n*=12. Data are expressed as mean±s.e.m.

The transgenerational transmission of high incentive motivation for cocaine could be attributed to pre-existing genetic variations or to acquired heritable epigenetic modifications. To dissociate the two possible mechanisms, we mated naive rats first to generate F1 offspring, and one week after mating, subjected the mated males to cocaine self-administration to identify Addict and Non-addict rats according to their performance (Fig. 7a). The offspring sired by Addict and Non-addict F0 rats before their exposure to cocaine were designated as Pre-Addict F1 and Pre-Non-addict F1 rats. In contrast to what we have observed in descendants of F0 rats mated after drug exposure (Fig. 1d), Pre-Addict F1 and Pre-Non-addict F1 rats exhibited no difference in cocaine self-administered or motivation for cocaine in PR test (Fig. 7b). No differences were observed in the animals' performance in the open field or the sucrose acquisition and motivation between these two groups (Supplementary Figs. 3a,b). Moreover, cocaine intake and motivation were similar in the mated and non-mated rats (Supplementary Fig. 3c). These data, combined with the results of yoke and intraperitoneal administration experiments, suggest that transgenerational inheritance of high incentive motivation for cocaine is an acquired trait that involves non-genetic mechanisms.

### Sperm methylome difference is maintained across generations

**Figure 8:**
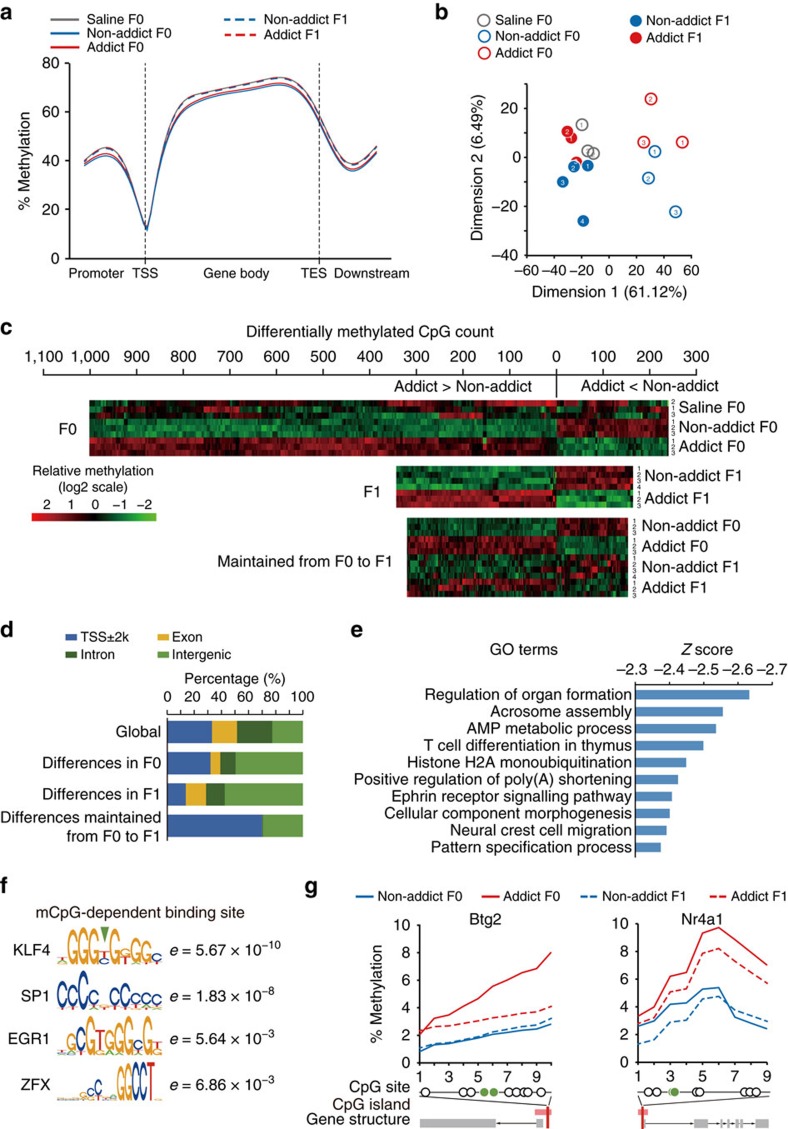
Identification of maintained DNA methylation alterations in the sperm of Addict F0 and F1 rats. (**a**) The average methylation levels of CpG sites upstream of the transcription start site (TSS), within the gene body, and the downstream of transcription termination sites (TES) in all genes detected. Addict F0 and Non-addict F0 showed lower global methylation level than Saline F0. (**b**) Euclidean distance-based multidimensional scaling indicated segregation of generation and addiction state. Number in circle refers to subject number in each group, corresponding to the subject number in **c**. (**c**) Methylation in 1,244 CpGs was significantly different between Addict F0 and Non-addict F0, and 522 differentially methylated CpGs were detected in Addict F1 versus Non-addict F1. The differential methylation was maintained in 475 CpGs across F0 to F1 generation. FDR≤0.05 was considered significant. (**d**) CpG distributions in TSS±2,000 bp area, exons, introns, and other intergenic elements. CpG methylation differences maintained from F0 to F1 were enriched in TSS±2,000 bp area. Differences maintained from F0 to F1/Global, odds ratio (95% confidence intervals), 273.95 (55.82-1340.73). (**e**) Ontology analysis of CpGs (in TSS±2,000 bp region) with differential methylation maintained from F0 to F1. Z scores from Enrichr were acquired and ranked. (**f**) Motif enrichment analysis of TSS±2,000 bp region by rGADEM and MotIV. The results revealed specific enrichment of KLF4 binding sites (*e*=5.67 × 10^−10^). (**g**) Methylation profiles of representative CpGs within differential methylation region (DMR) of Klf4-regulated genes. DMRs were calculated by BiSeq. Group averages are plotted. Btg2: DMR, Chr13: 55970169–55970230, three-way RM ANOVA, *F*_addict × generation × site_ (9, 81)=7.863, *P*=3.03 × 10^−8^; Bonferroni *post hoc*, *P*=3.5 × 10^−14^, Non-addict versus Addict. Nr4a1: DMR, Chr7: 140712984–140713087; three-way RM ANOVA, *F*_addict × generation × site_ (8, 72)=0.832, *P*=0.58; Bonferroni *post hoc*, *P*=1.1 × 10^−11^, Non-addict versus Addict. Non-addict F0, *n*=3; Addict F0, *n*=3; Non-addict F1, *n*=4; Addict F1, *n*=3.

DNA methylation, a stable epigenetic modification that regulates gene transcription and splicing, may be one of possible mechanisms for transgenerational inheritance of acquired traits^20^. We therefore performed reduced-representation bisulfite sequencing (RRBS) to assess DNA methylation-enriched sites in sperm samples from Saline F0, Addict F0, Non-addict F0, Addict F1, and Non-addict F1. Sperm samples of 3–4 rats per group were individually sequenced at 20M-read depth. In the 16 samples sequenced, total 1,570,110 CpGs were detected in more than 6 samples and with ≥5 × coverage. We first calculated the associations of generation (F0,F1) and phenotype (Non-addict, Addict, Saline) traits with the global methylation profiles. Moderate general demethylation across all genetic elements analysed was detected in both Addict F0 and Non-addict F0 sperms, compared with Saline F0, likely due to the effect of cocaine intake; and there was also difference between Addict and Non-addict groups, indicating possible association of DNA methylation with incentive motivation for cocaine (Fig. 8a).

Multidimensional scaling revealed clear segregation of F0 with F1 generations in Dimension 1, correlating with the global demethylation caused by direct drug exposure, and separation of Addict and Non-addict groups in Dimension 2 (Fig. 8b), Notably, Fisher's exact test also indicate associations of generation and phenotype traits (generation, *P*=4.0 × 10^−5^; phenotype, *P*=4.0 × 10^−5^; generation × phenotype, *P*=0.015).

Next, we sought to find the methylation differences, if any, that might be relevant to the transgenerational inheritance. Therefore, we identified CpG sites that were differentially methylated between Addict versus Non-addict F0 and maintained across generations. We found that methylation at 1,244 CpG sites was significantly different between Addict and Non-addict F0 (Fig. 4c, upper panel, *P*<0.05) and 522 CpG sites were differentially methylated in their F1 offspring (Fig. 8c, middle panel). Importantly, 475 differentially methylated CpG sites were maintained in F0 and F1 generation (hereafter referred to as Maintained Differences, Fig. 8c, lower panel). Maintained methylation differences were enriched in hypo-methylated regions (maximum methylation level ≤0.3, 93.7% in CpGs with Maintained Differences versus 34.2% in CpGs commonly covered) and also featured in TSS±2,000 bp distribution (Fig. 8d; 66.5% in CpGs with Maintained Differences versus 34.8% in CpGs commonly covered), indicating their potential involvement in transcriptional modulation of promoter region. Ontology analysis of genes with Maintained Differences in TSS±2,000 bp region also indicate that GO terms associated with development and morphology are overrepresented (Fig. 8e and Supplementary Table 1). Interestingly, binding motifs for transcription factors, including KLF4, SP1, EGR1 and ZFX, were enriched in these regions (Fig. 8f). Transcription activity of KLF4, which has the most significant e-value, has been demonstrated to be regulated by methylation level^21^. KLF4 binding sites were detected in promoters of *Btg2* and *Nr4a1*, and they exhibited preserved higher methylation in Addict groups than Non-addict groups (Fig. 8g). This result was consistent with previous report that both *Btg2* and *Nr4a1* regulate neurogenesis and other brain functions via transcriptional mechanisms^22^,^23^.

To provide insight into potential mechanistic link of sperm DNA methylation changes to descendant brain functions, we performed transcriptome sequencing of Non-addict F1 and Addict F1 in the nucleus accumbens (NAc). 292 genes exhibit higher expression level in Non-addict F1 than Addict F1 (fold change ≥2, *P*<0.05), around 3.5-fold of the number downregulated, coincides with lower DNA methylation level in sperms of Non-addict groups than Addict groups (Supplementary Fig. 4a). Although it is well known that DNA methylation profiles change dynamically during development, and differs from sperm to brain, we found that differential promoter methylation in F1 sperm significantly collapsed with alterations of gene expression in adult NAc (Supplementary Fig. 4b). Forty-nine of the 292 genes, namely *Gabrd*,*Chat*,*Kcnn3* showed transcriptional differences in F1 of Addict versus Non-addict groups, consistent with DNA methylation differences in sperm maintained from F0 to F1 (Supplementary Figs. 4c, d). The differentially methylated and expressed genes from RRBS and RNA-sequencing (RNA-seq) data sets were compared, and a functionally grouped annotation network was deduced. As shown in Supplementary Fig. 4e, genes that differential methylated between Addict and Non-addict groups were enriched in functions that regulate developmental process, consistent with the result shown in Fig. 8e. Several pathways, such as EGFR regulation, gap junction, and axonal guidance pathways, were co-enriched in methylation and transcription data, but in a co-clustering pathway, often distinct genes from methylation and transcription datasets were involved. Network analysis of genes from these clusters reveals possible signalling network connections between genes enriched in methylation and expression. As shown in Supplementary Fig. 4f, ephrins and EPH-related receptors (EPHA5, EFNA1), SHH, ISL1 and TBX5, which strictly controls embryonic development and were differentially methylated in sperm, regulate key plasticity-regulators, such as BDNF, JUN, and Tubulin family members. These downstream molecules were enriched in transcriptome data.

## Discussion

Familial aggregation of drug addiction has been implicated by epidemiological studies^10^,^11^. Extensive studies have been focused on the identification of genetic variations that are associated with risk of developing drug addiction, and genome-wide linkage and association studies have revealed many genes and genetic variants associated with drug response and related behaviour^24^,^25^,^26^,^27^. Significant intergenerational effects of maternal substance exposure during pregnancy on the development and behaviour of offspring have been reported in many experimental studies. Epidemiological data suggests attention deficit, hyperactivity disorder and learning disabilities^28^ in children with prenatal cocaine experience. Enhanced cocaine self-administration^29^, blunted drug sensitivity^30^ and developmental delays^31^ were observed in animal models. Intergenerational effects of prior cocaine experience transmitted via germline have received recent attention. It was reported that maternal cocaine exposure before pregnancy can serve to enhance psychomotor sensitivity to cocaine in offspring^32^. Paternal cocaine use causes intergenerational cocaine-resistant phenotype, anxiety^33^, depression-like behaviour^34^, impaired attention and working memory^35^. Experimentation is still needed to explain the apparent contrast of subtle behaviour changes in animal models with the high liability in human epidemiological studies.^36^ Studies supporting non-Mendelian heritable factors that may predispose offspring to drug addiction are lacking. In the current study, we used a rat model of cocaine self-administration and showed that high incentive motivation elicited by voluntary cocaine administration could be transmitted to subsequent generations. We also show that the transgenerational inheritance effect is not caused by pre-existing genetic variations, but rather it is an acquired trait that is induced by highly motivated drug-seeking experience.

Furthermore, our data indicated that the effect of non-contingent drug intake and reinforcing properties of cocaine on offspring are distinct. Findings by Vassoler *et al*.^19^, together with the present data, demonstrate that the paternal exposure to cocaine results in reduced cocaine seeking behaviour. Drug exposure per se acts as a novel chemical exposure, leading to protective or deleterious effects in offspring in some addiction-resilient individuals, such as drug-resistance^19^, increased anxiety level^34^ and impaired learning and memory^37^, raising concerns on the potential adverse effects of using psychostimulant, and perhaps other drugs. Moreover, we found that it is high motivation for cocaine reinforcement elicited by voluntary drug self-administration, not the total intake of addictive drug itself, that contributes to transgenerational inheritance of high incentive motivation for cocaine, implicating a key role of high responding to incentive reward induced by addictive drugs in the transgenerational inheritance. Our results suggest that highly motivated drug seeking experience may increase the vulnerability to drug reinforcement in descendants, and/or decrease resistance to drug addiction. It is possible that under certain circumstance, the decrease in resistance to drug addiction or absence of protection, may render offspring of Addict animals more vulnerable to cocaine addiction.

Emerging evidence suggests that epigenetic mechanisms, regulated by environmental stimuli, allow individuals to adapt to the dynamic changes in the surrounding environment^38^,^39^,^40^, including changes in nutritional and emotional status^41^,^42^. Studies indicate that gamete epigenomic information, including DNA methylation, chromatin patterning, noncoding RNAs and mitochondria, is vital to transgenerational inheritance of acquired phenotypes^20^. In the present study, we observed segregation in DNA methylation patterns in the sperm of Addict and Non-addict F0 rats and their F1 descendants, and identified regions that exhibit maintained differential methylation in F0 and F1, providing an epigenetic link to transcriptomic changes of addiction-related signalling pathways in offspring NAc. Our study demonstrated that highly motivated cocaine-seeking experience induced DNA methylation alterations different from that induced by the intake of cocaine itself, and such epigenetic alterations could be transmitted to subsequent generations, in parallel with transgenerational inheritance of high responding to drug reward. These observations support an epigenetic mechanism for the transgenerational inheritance of incentive motivational properties for cocaine, which includes, but is not limited to DNA methylation. Although the contribution of sperm DNA methylation to descendant neuronal function is not clear, we speculate that so-called epigenetic engrams^43^ of addiction-like behavioural experience in previous generations may induce adaptation of brain functions in offspring to facilitate prompt and favourable adaptive responses on their exposure to cocaine. However, factors that contribute to individual variabilities of motivational response to cocaine remain to be understood.

## Methods

### Animals and housing

All experimental naive Sprague-Dawley rats (Shanghai Center of Experimental Animal, Chinese Academy of Sciences) and their descendants were housed at 23 °C with a 12 h reverse dark/light cycle (on 20:00 hours, off 08:00 hours). They were allowed free access to food and water before behavioural experiments and housed in groups (3–4 rats per cage) before surgery. Room humidity was controlled at 40%. All the behavioural experiments were carried on using male rats aged 8–12 weeks. F0 rats were habituated for at least 2 weeks before experimentation. All animal treatments were strictly in accordance with the National Institutes of Health Guide for the Care and Use of Laboratory Animals and were approved by Animal Care and Use Committee of Shanghai Medical College of Fudan University.

### Food training and surgery

To facilitate the rats to self-administer cocaine, rats 8–10 weeks old were food-restricted and maintained at 85% of their ad libitum body weight 3 days before, and were then trained to press the active lever to get 45 mg food pellets in the operant chambers (Med-Associates) for 7 days. The training procedure was FR1 (one lever press/pellet) for 5 days and then FR5 (five lever presses/pellet) for 2 days. The rats which met the criteria of obtaining 100 food pellets per FR5 session were subjected to surgery. These rats were anaesthetized with chloral hydrate (0.4 g kg^−1^, Sangon) intraperitoneally. We positioned a silastic catheter about 3 cm into the right jugular vein and attached the other end with a stainless steel pedestal. The pedestal was then mounted to the rat's skull by dental cement. The rats were allowed to recover for 7 days after surgery. The catheters were flushed daily with 0.1 ml saline containing heparin (30 IU ml^−1^) and gentamycin (0.5 mg ml^−1^).

### Intravenous cocaine self-administration

After recovery, rats were allowed to self-administer cocaine in a daily 4 h FR session. When the rat presses the active press, an injection of cocaine (Qinghai Pharmaceutical Firm) at 0.75 mg kg^−1^ per injection over 4 s was presented and accompanied by a conditioned cue, including the illumination of the stimulus light and an audible tone for 20 s. The press on the inactive lever had no programmed consequences. In our procedure, the rats were first trained under the FR1 program for 5 days, then FR3 for 2 days and FR5 for 5 days. Then the program was changed to a 6 h progressive-ratio (PR) schedule, in which response requirements for each successive injection increased by progressive increments. The response requirement for each injection (i) follows i^th^injection=Int(5e^0.25i-5^) and the session stops when the rat takes >1 h to satisfy the response requirement. The number of lever presses for the last injection in the PR schedule was defined as the break point. The catheter patency was verified after PR schedule by anaesthesia with chloral hydrate and the data of rats with catheter problem was excluded. Lever presses during FR5 and break point of individual rats were scored (x) as before^44^, following 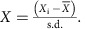 *X*_i_ is the behaviour value for each rats, 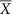 is the mean behaviour value for all the rats, and s.d. is the s.d. of the population behaviour value. Twenty-four hours after the last self-administration session, each male rat was housed with two naive female rats to generate F1. F2 rats were sired by crossing naive F1 with two naive female rats.

### Sucrose and food self-administration

The rats were initially maintained at 85% original body weight for 3 days before the training and trained to press the active lever to get a 45 mg sucrose pellet or grain-based food pellet (Bio-Serv) under FR1 program for 5 days, then FR5 for 2 days and switched to PR schedule.

### Dose-response effect on locomotor activity

Rats were habituated to the locomotor chamber for one hour, and were then administered hourly ascending dose of cocaine (0, 2.5, 7.5, 15, 25 mg kg^−1^, intraperitoneal). Locomotor activity was measured during each hour of the testing period.

### Open field test

Locomotor activity was measured using commercial open field activity chambers for rats (Med-Associates). The session lasted for 30 min. Data collection and analysis were done using Med Associates Activity Monitor program.

### Cocaine intraperitoneal injection

Naive male SD rats were injected intraperitoneally with cocaine (15 mg kg^−1^) or equivalent volume of saline every 12 h for 7 days, and were immediately returned to their home cage after each injection. Twenty-four hours after the last injection, each male rat was housed with two naive female rats to generate F1. F2 rats were sired by crossing naive F1 with two naive female rats.

### Sperm collection and purification

Male rats were euthanized with chlorohydrate overdose. The testes and cauda epidymides were dissected, washed with PBS and placed in 35 mm petri dishes containing HBSS supplemented with 20 mM HEPES pH 7.2, 1.2 mM MgSO_4_·7H_2_O, 1.3 mM CaCl_2_·2H_2_O, 6.6 mM sodium pyruvate, 0.05% lactate, 2 mM GlutaMAX Supplement (Gibco) and 0.5% BSA^45^. Released sperm-containing supernatant was moved to 5 ml collection tubes and let stand for 10 min at 37 °C for swimming up. Supernatant was further filtered through 40 μm nylon mesh and laid on top of a three-layer gradient of 80, 60 and 30% Percol, centrifuged for 10 min at 1,000*g* at room temperature. Sperms were collected from 60% layer of the gradient, washed with the incubation buffer and cryopreserved. DNA was extracted from purified sperm according to the protocol described by Griffin^46^ and then preserved in TE buffer.

### Reduced representation bisulfite sequencing

Library preparation was performed by BasePair BioTechnology. The quality and integrity of DNA samples were controlled by A260/280 ratio (≥1.8) and agarose gel electrophoresis. For library preparation, 1 μg genomic DNA was digested by MspI to generate short fragments that contain CpG dinucleotides at the ends. Then digested DNA was end-repaired, A-tailed and ligated to methylated Illumina adapters following the user manual of Truseq DNA Sample Prep Kit (Illumina, USA) Then, the CpG-rich DNA fragments (160–340 bp) are size selected, subjected to bisulfite conversion by MethylCode Bisulfite Conversion Kit (Life Technologies, USA), and PCR amplified by ZymoTaq PreMix (Zymo, USA). The libraries were sequenced 125 bp PE on Illumina Hiseq 2500. At least three biological replicates were sequenced for each condition.

### RNA sequencing

Total RNA was extracted from NAc of drug- and experiment- naive Addict and Non-addict F1 rats with standard Trizol method. RNA from three rats per group was pooled and subjected to RNA integrity analysis and subsequent library preparation procedures with TruSeq RNA Library Prep Kit (Illumina, USA). 100-bp PE reads were generated with Illumina Hiseq 2000.

### Sequencing data analysis

The raw RRBS sequencing reads were quality checked and trimmed with Trim Galore (http://www.bioinformatics.babraham.ac.uk/projects/trim_galore/) for low quality (Phred score <30) nucleotides, adapters and artificially introduced bases in the end-repair step. Reads too short (<20 bp) were filtered. Alignment and methylation calling of the filtered reads were then performed with Bismark^47^ using genome assembly for Rattus norvegicus UCSC (rn5, RefSeq gene annotations). Parameters used were default except that 3 maximum mismatches in the ‘seed' were allowed and ‘paired-end' was specified. Bowtie^48^ short read aligner was used. The bisulfite conversion rate was estimated by calculating the C to T conversion at non-CpG sites. Differential methylation region was calculated with BiSeq^49^. DMRs were considered significant with *P*<0.05. rGADEM^50^ was used for unseeded motif analysis with default settings and MotIV for e value calculation and visualization based on JASPAR 2010 scores. Euclidean-based multidimensional scaling and DNA methylation profiles across genes were plotted with RnBeads^51^. Expression in heatmap were clustered based on Euclidean distances with R package ‘pheatmap'. RNA-seq data was aligned to rn5 and calculated for differential expression with the Tuxedo suite^52^,^53^ using default settings. ClueGO was used to construct network relationships of genes and pathways from RRBS and RNA-seq data set^54^.

### Western blotting

Drug- and experiment- naive F1 rats from Saline, Non-addict-SA, Non-addict-Yoke, Addict-SA and Addict-Yoke groups were killed. Brains were dissected immediately, and mPFC was collected and homogenized on ice in 200 μl of lysis buffer (10 mM Tris·HCl, pH 7.4, 0.5% Triton X-100, 0.5% Nonidet P-40, 50 mM NaCl, 1.0 mM EDTA, 50 mM NaF, 100 μM Na_3_VO_4_, 1.0 μM PMSF and protease inhibitors). After 30-min incubation on ice, lysate was centrifuged at 16,000 × g for 10 min and supernatant was collected. Bradford assay was used for protein level normalization. Samples were boiled in SDS/PAGE loading buffer (0.1 mM Tris·HCl, pH 6.8, 5% glycerol, 1.5% SDS, 5% β-mercaptoethanol, and 0.002% bromophenol blue) for 5 min. For each sample, 30 μg total protein was loaded and separated by 18% SDS–PAGE. Blots were probed with primary antibody at 4 °C overnight: anti-BDNF (ab6201, Abcam, 1:100), and anti-β-actin (A2066, Sigma-Aldrich, 1:2,000). Blots were then incubated with IRDye 800CW-conjugated or 700CW-conjugated secondary antibodies (Rockland Biosciences). Infrared fluorescence images were obtained with the Odyssey infrared imaging system (Li-Cor Bioscience). Quantification was performed with Image Studio Lite Ver 5.2. The blotting experiments were repeated three times and average expression of each sample was used. BDNF level was normalized by comparing with the coordinate β-Actin expression level. Uncropped image for the representative plot is provided in Supplementary Fig. 5.

### Statistical analysis

Behavioural data in cocaine self-administration, sucrose self-administration during FR program was analysed with mixed linear model with repeated measurements (MMRM). Cocaine dose induced locomotion were analysed using two-way repeated-measures analysis of variance (ANOVA) followed by Bonferroni *post hoc* tests, break point was analysed by Mann–Whitney Rank-Sum Test or Friedman two-way ANOVA test by ranks. The open field test and real-time PCR data were analysed by Student's *t* test. Sample size estimation was conducted on alpha value of 0.05 and desired power of 0.80. *P*<0.05 was considered statistically significant. Data are presented as mean±s.e.m.

### Data availability

Raw sequencing data that support the findings of this study have been deposited in the Gene Expression Ominibus with accession code GSE72401 (http://www.ncbi.nlm.nih.gov/geo/query/acc.cgi?acc=GSE72401). The authors declare that all other data supporting the findings of this study are available within the manuscript and its supplementary files or are available from the corresponding author on request.

## Additional information

**How to cite this article:** Le, Q. *et al*. Drug-seeking motivation level in male rats determines offspring susceptibility or resistance to cocaine-seeking behaviour. *Nat. Commun.* **8,** 15527 doi: 10.1038/ncomms15527 (2017).

**Publisher's note:** Springer Nature remains neutral with regard to jurisdictional claims in published maps and institutional affiliations.

## Supplementary Material

Supplementary Information Supplementary Figures and Supplementary Table
