# Supplementary material for: Drug-seeking motivation level in male rats determines offspring susceptibility or resistance to cocaine-seeking behaviour
Source: Nat Commun. 2017 May 30;8:15527. doi: 10.1038/ncomms15527 (PMC5459992; doi:10.1038/ncomms15527)
Supplement: Supplementary Information — Supplementary Figures and Supplementary Table [file ncomms15527-s1.pdf]

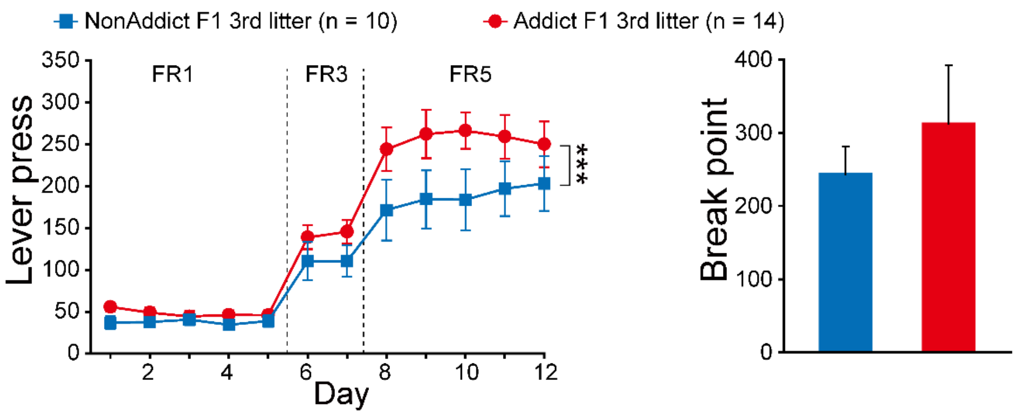

Supplementary Fig. 1 The third litter from Addict F0 showed higher cocaine consumption compared with the third litter from NonAddict F0 rats. The Addict F1 (3rd litter) showed higher cocaine intake than NonAddict F1 (3rd litter) in FR5 schedule, but no difference in motivation measured in PR schedule. Lever press, Group × FR, MMRM, χ^2 (2) = 22.56, $P < 0.001$; Group, $P = 0.109$; FR5, $^{**}P = 0.004$, NonAddict F1 (3rd litter) vs. Addict F1 (3rd litter). Break point, Mann-Whitney Rank Sum Test, $Z = 0.383$, $P = 0.702$. NonAddict F1 3rd litter, n = 10; Addict F1 3rd litter, n = 14. Data are expressed as mean ± s.e.m..

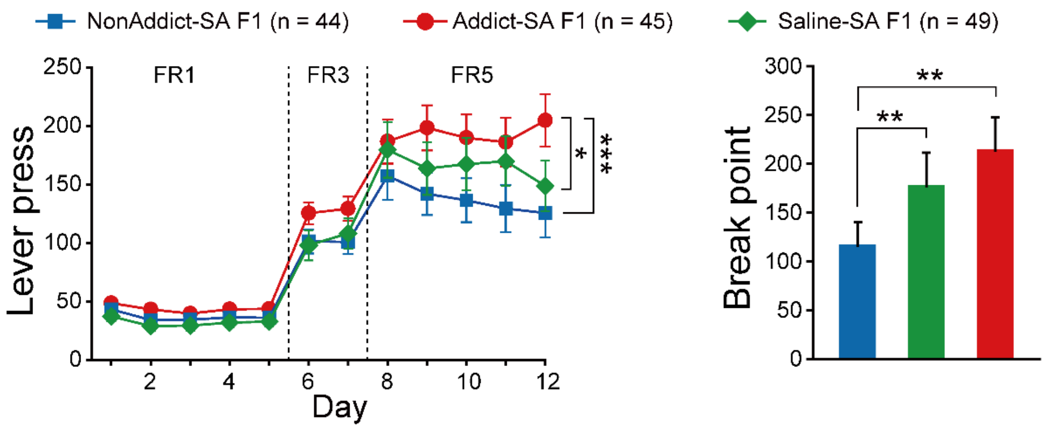

Supplementary Fig. 2 Susceptibility and resistance to cocaine-seeking behaviour in Addict and NonAddict F1. Cocaine intake and motivation data of Addict F1, NonAddict F1, and Saline F1 rats. Lever press, Group × FR, MMRM, $\chi 2$ (4) = 23.72, *P* = 0.0001; Group, *p* = 0.117; FR5, **P* = 0.027, Addict F1 vs. Saline F1, ****P* < 0.001, Addict F1 vs. NonAddict F1. Break point, Kruskal-Wallis one-way ANOVA on ranks, *H* (2) = 13.676, *P* = 0.0011; Two-sample Wilcoxon rank-sum, ***P* = 0.0011 NonAddict F1 vs. Addict F1, ***P* = 0.0026, NonAddict F1 vs. Saline F1. NonAddict F1, n = 44; Addict F1, n = 45; Saline F1, n = 49. Data are expressed as mean ± s.e.m..

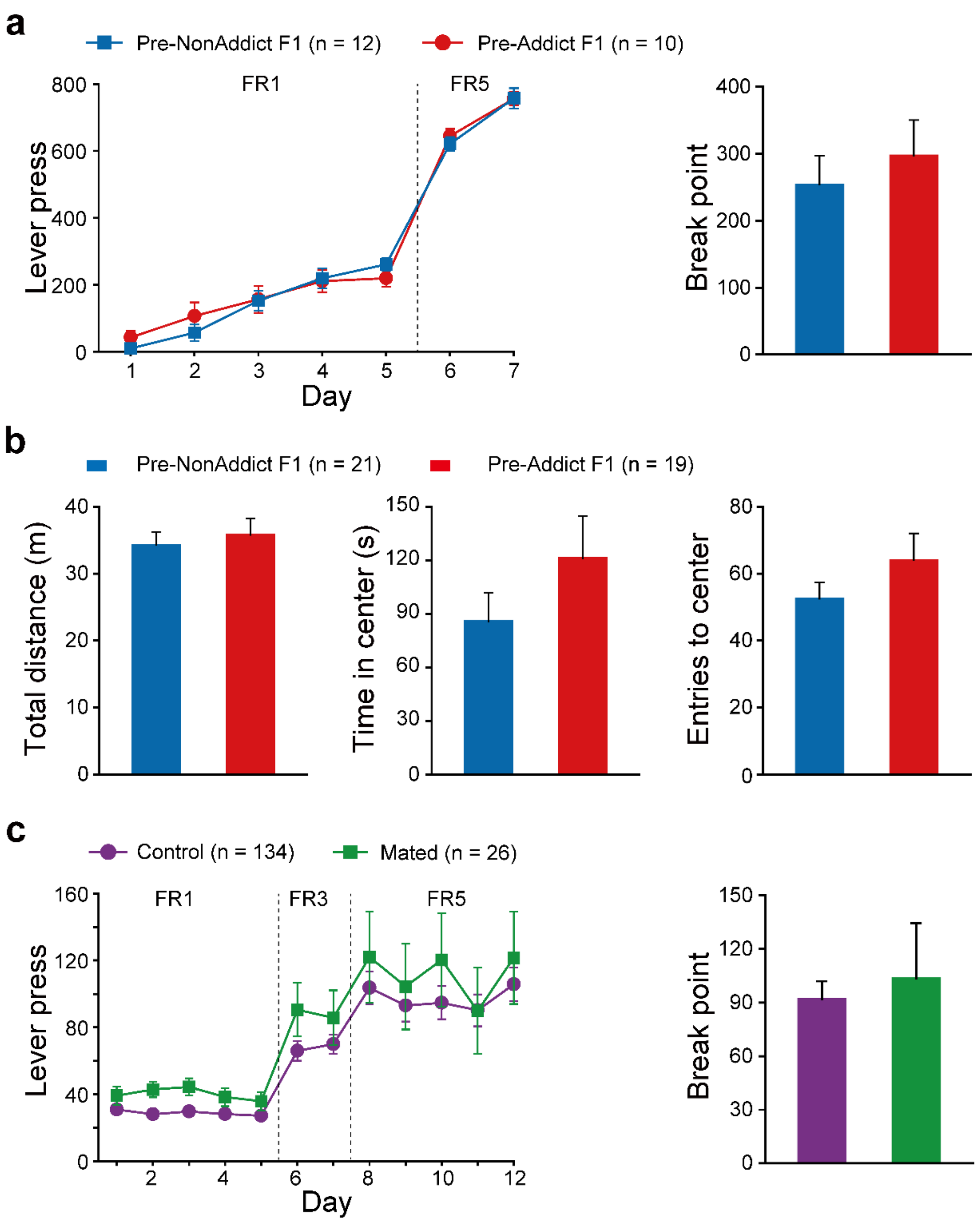

Supplementary Fig. 3 Pre-Addict F1 and Pre-NonAddict F1 rats exhibited no significant difference in open field and sucrose self-administration tests. (a) Sucrose self-administration test. Lever press, Group × FR, MMRM, χ2 (1) = 0.01, *P* = 0.924. Break point, Mann-Whitney Rank Sum Test, *Z* = 0.635, *P* = 0.526. Pre-NonAddict F1, n = 12; Pre-Addict F1, n = 10. (b) Open field test. Total distance, Student's *t*-test, *t* (38) = 0.488, *P* = 0.628. Time in center, Student's *t*-test, *t* (38) = 1.271, *P* = 0.212. Entries to center, Student's *t*-test, *t* (38) = 1.254, *P* = 0.218. Pre-NonAddict F1, n = 21; Pre-Addict F1, n = 19. (c) There was no difference observed in lever press or break point for cocaine between Mated and Control rats. Lever press, Group × FR, MMRM, χ^2 (2) = 0.71, *P* = 0.701; Group, *P* = 0.237; FR5, *P* = 0.296. Break point, Mann-Whitney Rank Sum Test, *Z* = 0.090, *P* = 0.928. Mated, n =26; Control, n = 114. Data are expressed as mean ±s.e.m..

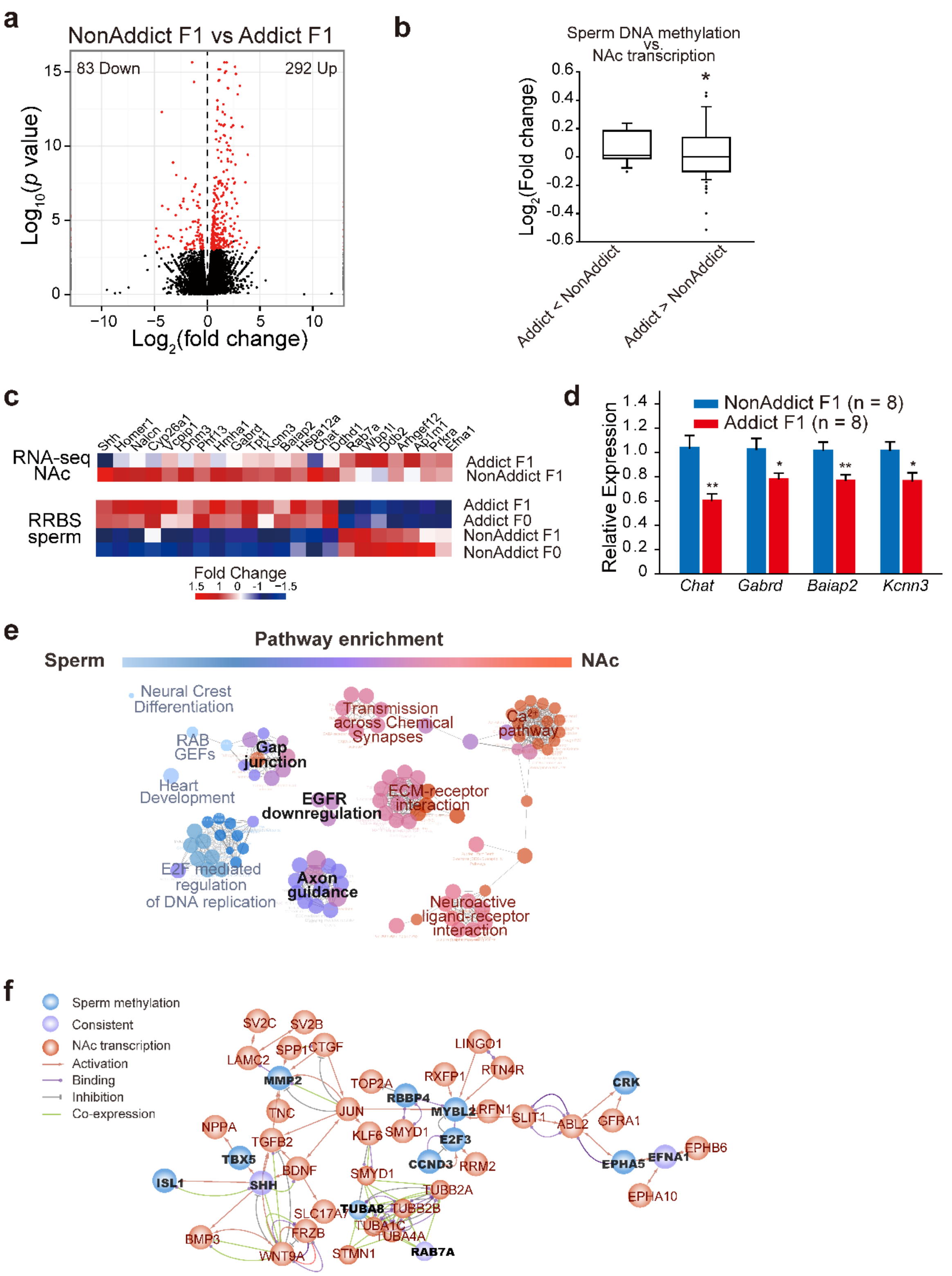

Supplementary Fig. 4 Comparative analysis of sperm DNA methylome and adult NAc transcriptome datasets of Addict and NonAddict rats. (a) “Volcano plot” of statistical significance against fold-change of NonAddict F1 vs. Addict F1. Red dots represent differentially expressed genes with statistical significance (fold change ≥ 2, $P < 0.05$). (b) Comparison of methylation and transcription levels. Genes with Maintained CpGs in TSS ±2,000 bp region were divided according to DNA methylation levels into Addict < NonAddict and Addict > NonAddict groups, and fold-change in gene expression of NonAddict F1 vs. Addict F1 was plotted. Data are expressed as median ± s.d.. Mann-Whitney Rank Sum Test, $Z = 5402$, $*P = 0.027$. (c) Heatmap of genes with transcriptional differences in F1 NAc consistent with methylation differences in sperm maintained from F0 to F1. Methylation level is represented by mean of DNA methylation in gene-associated region in each group. (d) Expression validation by qRT-PCR. Data are expressed as mean ± s.e.m.. Student’s *t*-test, $*P < 0.05$, $**P < 0.01$, NonAddict F1 vs. Addict F1. Data are expressed as mean ± s.e.m.. (e) Pathway clustering of differentially methylated (blue)/expressed (red) genes. Intermediate colors represent pathways enriched in both methylation and transcription datasets. (f) Gene regulatory network derived from co-clustering networks.

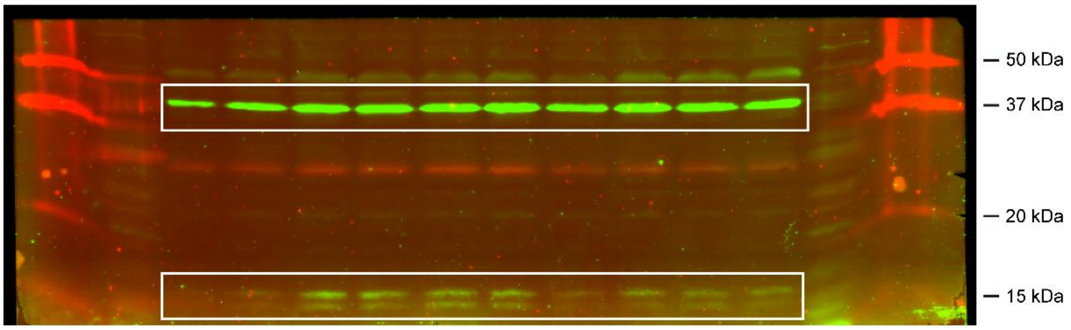

Supplementary Fig. 5 Uncropped scans for Western blot data in main figures.

Supplemental Table 1. The distribution of Maintained CpGs within gene proximal regions.

ENSEMBL Number	Symbol	Description	Position	Width	Promoter	Exons	Intron	TSS±2k
ENSRNOG00000014382	C2cd5	C2 calcium-dependent domain containing 5	Chr4: 241536427 - 241536501	75	Y			Y
ENSRNOG00000004049	Baiap2	BAI1-associated protein 2	Chr10: 108706634 - 108706702	69	Y			Y
ENSRNOG00000003300	Btg2	BTG family, member 2	Chr13: 55970169 - 55970230	62	Y			Y
ENSRNOG00000021109	Ccdc114	coiled-coil domain containing 114	Chr1: 102990394 - 102990447	54		Y		
ENSRNOG00000007607	Nr4a1	nuclear receptor subfamily 4, group A, member 1	Chr7: 140713076 - 140713126	51	Y			Y
ENSRNOG00000014071	Ddb2	damage specific DNA binding protein 2	Chr3: 86761951 - 86761999	49	Y			Y
ENSRNOG00000046002	LOC301124	hypothetical LOC301124	Chr9: 9444016 - 9444064	49			Y	Y
ENSRNOG00000020020	Wbp1l	WW domain binding protein 1-like	Chr1: 273788985 - 273789032	48	Y			Y
ENSRNOG00000025792	Crk	v-crk avian sarcoma virus CT10 oncogene homolog	Chr10: 64174982 - 64175028	47			Y	Y
ENSRNOG00000017158	Eif4g2	eukaryotic translation initiation factor 4, gamma 2	Chr1: 182879856 - 182879901	46			Y	Y
ENSRNOG00000001049	Tpt1	tumor protein, translationally-controlled 1	Chr15: 61598789 - 61598833	45		Y		Y
ENSRNOG00000011114	Tbpl1	TBP-like 1	Chr1: 25442093 - 25442135	43		Y		Y
ENSRNOG00000017468	Trappc6a	trafficking protein particle complex 6A	Chr1: 81681765 - 81681804	40	Y			Y
ENSRNOG00000003134	Slc4a4	solute carrier family 4, sodium bicarbonate cotransporter, member 4	Chr14: 20723876 - 20723915	40	Y			Y
ENSRNOG00000006980	Vcpip1	valosin containing protein (p97)/p47 complex interacting protein 1	Chr5: 14031012 - 14031051	40	Y			Y
ENSRNOG00000039076	LOC688452,Mir125a,Mir99b,Mirlet7e	hypothetical protein LOC688452,microRNA 125a,microRNA 99b,microRNA let-7e	Chr1: 60623939 - 60623975	37	Y			Y
ENSRNOG00000015953	Oaz2	ornithine decarboxylase antizyme 2	Chr8: 70899552 - 70899587	36		Y		Y
ENSRNOG00000018019	Hspa12a	heat shock protein 12A	Chr1: 287377633 - 287377667	35			Y	Y
ENSRNOG00000026799	Zbtb9	zinc finger and BTB domain containing 9	Chr20: 7625556 - 7625589	34	Y			Y
ENSRNOG00000004752	Nalcn	sodium leak channel, non-selective	Chr15: 113454895 - 113454927	33			Y	Y
ENSRNOG00000008095	Onecut1	one cut homeobox 1	Chr8: 81386733 - 81386764	32			Y	Y
ENSRNOG00000016385	Gabrd	gamma-aminobutyric acid (GABA) A receptor, delta	Chr5: 176267644 - 176267669	26	Y			Y
ENSRNOG00000001134	Rfc5	replication factor C (activator 1) 5	Chr12: 46752419 - 46752440	22			Y	Y
ENSRNOG00000018116	Kcna3	potassium voltage-gated channel, shaker-related subfamily, member 3	Chr2: 229233525 - 229233546	22		Y		Y
ENSRNOG00000033787	Adamtsl5	ADAMTS-like 5	Chr7: 12398396 - 12398416	21	Y			Y
ENSRNOG00000008639	Pabpc1	poly(A) binding protein, cytoplasmic 1	Chr7: 75570181 - 75570201	21	Y			Y
ENSRNOG00000007573	Hoxb9	homeo box B9	Chr10: 83922970 - 83922989	20		Y		Y
ENSRNOG00000019682	Timm13	translocase of inner mitochondrial membrane 13 homolog (yeast)	Chr7: 11845175 - 11845193	19			Y	Y
ENSRNOG00000012297	42071	membrane-associated ring finger (C3HC4) 8, E3 ubiquitin protein ligase	Chr4: 214232962 - 214232979	18		Y		Y
ENSRNOG00000007805	Mybl2	myeloblastosis oncogene-like 2	Chr3: 165617381 - 165617397	17		Y		Y

ENSRNOG00000009102	Fermt2	fermitin family member 2	Chr15: 23839845 - 23839860	16		Y		Y
ENSRNOG00000000505	Rpl10a	ribosomal protein L10A	Chr20: 10109132 - 10109147	16		Y		Y
ENSRNOG00000013220	Hmha1	histocompatibility (minor) HA-1	Chr7: 12771481 - 12771496	16			Y	
ENSRNOG00000020027	Arhgef2	rho/rac guanine nucleotide exchange factor (GEF) 2	Chr2: 207396192 - 207396206	15	Y			Y
ENSRNOG00000011195	Prkra	protein kinase, interferon inducible double stranded RNA dependent activator	Chr3: 70080907 - 70080921	15		Y		Y
ENSRNOG00000007673	Ppig	peptidylprolyl isomerase G	Chr3: 62571976 - 62571989	14			Y	Y
ENSRNOG00000050258	Ccnd3	cyclin D3	Chr9: 14332072 - 14332085	14		Y		Y
ENSRNOG00000012247	Rab7a	RAB7A, member RAS oncogene family	Chr4: 185205917 - 185205928	12	Y			Y
ENSRNOG00000009046	Phf13	PHD finger protein 13	Chr5: 172726055 - 172726066	12			Y	Y
ENSRNOG00000007503	Adam17	ADAM metallopeptidase domain 17	Chr6: 60316136 - 60316146	11	Y			Y
ENSRNOG00000020411	Sec23ip	SEC23 interacting protein	Chr1: 207214821 - 207214830	10		Y		Y
ENSRNOG00000020706	Kcnn3	potassium intermediate/small conductance calcium-activated channel, subfamily N, member 3	Chr2: 208266755 - 208266764	10	Y			Y
ENSRNOG00000023300,ENSRNOG00000025012	Chat,Grid1	choline O-acetyltransferase,glutamate receptor, ionotropic, delta 1	Chr16: 9495836 - 9495844	9	Y			Y
ENSRNOG00000031031	Zfp292	zinc finger protein 292	Chr5: 54931258 - 54931265	8		Y		Y
ENSRNOG00000002301	Uso1	USO1 vesicle transport factor	Chr14: 17354828 - 17354834	7	Y			Y
ENSRNOG00000012556	Isl1	ISL LIM homeobox 1	Chr2: 66884564 - 66884570	7	Y			Y
ENSRNOG00000048169	Tuba8	tubulin, alpha 8	Chr4: 220864699 - 220864705	7			Y	Y
ENSRNOG00000007477	Edn3	endothelin 3	Chr3: 178902916 - 178902921	6	Y			Y
ENSRNOG00000021492	Rbbp4	retinoblastoma binding protein 4	Chr5: 151256647 - 151256652	6	Y			Y
ENSRNOG00000005828	Skp1	S-phase kinase-associated protein 1	Chr10: 37368377 - 37368381	5			Y	Y
ENSRNOG00000012083	St6galnac2	ST6 (alpha-N-acetyl-neuraminyl-2,3-beta-galactosyl-1,3)-N-acetylgalactosaminide alpha-2,6-sialyltransferase 2	Chr10: 105331628 - 105331632	5	Y			Y
ENSRNOG00000019039	Inpp5e	inositol polyphosphate-5-phosphatase E	Chr3: 9217226 - 9217230	5	Y			Y
ENSRNOG00000050547	Syngr2	synaptogyrin 2	Chr10: 106452005 - 106452008	4		Y		Y
ENSRNOG00000023509	Irs2	insulin receptor substrate 2	Chr16: 83286984 - 83286987	4	Y			Y
ENSRNOG00000016695	Mmp2	matrix metallopeptidase 2	Chr19: 26658642 - 26658645	4		Y		Y
ENSRNOG00000000481	Cuta	cutA divalent cation tolerance homolog (E. coli)	Chr20: 7592065 - 7592068	4		Y		Y
ENSRNOG00000034025	Ptprj	protein tyrosine phosphatase, receptor type, J	Chr3: 86098721 - 86098724	4			Y	Y
ENSRNOG00000005964	Nr4a3	nuclear receptor subfamily 4, group A, member 3	Chr5: 68300503 - 68300505	3			Y	Y
ENSRNOG00000050145	Ahnak	AHNAK nucleoprotein	Chr1: 232122315 - 232122316	2	Y			Y
ENSRNOG00000042384	Kctd7	potassium channel tetramerization domain containing 7	Chr12: 31971421 - 31971422	2	Y			Y

ENSRNOG00000000693	Svop	SV2 related protein	Chr12: 50088883 - 50088884	2			Y	
ENSRNOG00000003716	Batf3	basic leucine zipper transcription factor, ATF-like 3	Chr13: 114308212 - 114308213	2	Y			Y
ENSRNOG00000017548	Fam53a	family with sequence similarity 53, member A	Chr14: 83064856 - 83064857	2		Y		Y
ENSRNOG00000050510	Rab1a	RAB1A, member RAS oncogene family	Chr14: 104208614 - 104208615	2		Y		Y
ENSRNOG00000011423	Pitx1	paired-like homeodomain 1	Chr17: 11049546 - 11049547	2		Y		
ENSRNOG00000011423	Pitx1	paired-like homeodomain 1	Chr17: 11049753 - 11049754	2		Y		
ENSRNOG00000012405	Tcf4	transcription factor 4	Chr18: 64603568 - 64603569	2			Y	
ENSRNOG00000020573	Efna1	ephrin A1	Chr2: 208011367 - 208011368	2	Y			Y
ENSRNOG00000007662	Zfp800	zinc finger protein 800	Chr4: 55269202 - 55269203	2	Y			Y
ENSRNOG00000022921	Dact2	dishevelled-binding antagonist of beta-catenin 2	Chr1: 57362383 - 57362383	1			Y	Y
ENSRNOG00000020618	Rpl13a	ribosomal protein L13A	Chr1: 102188826 - 102188826	1			Y	Y
ENSRNOG00000018391	Cdk2ap2	cyclin-dependent kinase 2 associated protein 2	Chr1: 226216262 - 226216262	1	Y			Y
ENSRNOG00000027491	Vldlr	very low density lipoprotein receptor	Chr1: 252487886 - 252487886	1			Y	Y
ENSRNOG00000016750	Cyp26a1	cytochrome P450, family 26, subfamily a, polypeptide 1	Chr1: 263865761 - 263865761	1		Y		Y
ENSRNOG00000020140	Pigq	phosphatidylinositol glycan anchor biosynthesis, class Q	Chr10: 15117634 - 15117634	1			Y	Y
ENSRNOG00000003050	Tbc1d9b	TBC1 domain family, member 9B (with GRAM domain)	Chr10: 35398215 - 35398215	1			Y	Y
ENSRNOG00000001399	Tbx5	T-box 5	Chr12: 43947486 - 43947486	1	Y			Y
ENSRNOG00000026490	Dnm3	dynamin 3	Chr13: 85271382 - 85271382	1			Y	Y
ENSRNOG00000004481	Adss	adenylosuccinate synthase	Chr13: 100386206 - 100386206	1		Y		Y
ENSRNOG00000002024	Epha5	EPH receptor A5	Chr14: 25418911 - 25418911	1			Y	Y
ENSRNOG00000009481	Ddhd1	DDHD domain containing 1	Chr15: 23995663 - 23995663	1			Y	Y
ENSRNOG00000001049	Tpt1	tumor protein, translationally-controlled 1	Chr15: 61598612 - 61598612	1	Y			Y
ENSRNOG00000025012	Chat	choline O-acetyltransferase	Chr16: 8853402 - 8853402	1			Y	
ENSRNOG00000014454	Ap1m1	adaptor-related protein complex 1, mu 1 subunit	Chr16: 19164597 - 19164597	1	Y			Y
ENSRNOG00000013751	Prosc	proline synthetase co-transcribed homolog (bacterial)	Chr16: 68849492 - 68849492	1		Y		Y
ENSRNOG00000012333	Kbtbd11	kelch repeat and BTB (POZ) domain containing 11	Chr16: 79276745 - 79276745	1		Y		Y
ENSRNOG00000014007	Gfod1	glucose-fructose oxidoreductase domain containing 1	Chr17: 25874109 - 25874109	1	Y			Y
ENSRNOG00000029273	E2f3	E2F transcription factor 3	Chr17: 37641687 - 37641687	1	Y			Y
ENSRNOG00000020129	Cdh3	cadherin 3	Chr19: 49538421 - 49538421	1			Y	Y
ENSRNOG00000015150	Spg7	spastic paraplegia 7 homolog (human)	Chr19: 66586490 - 66586490	1		Y		Y
ENSRNOG00000047014	Homer1	homer homolog 1 (Drosophila)	Chr2: 42106877 - 42106877	1	Y			Y
ENSRNOG00000000524	RGD735065	similar to GI: 13385412-like protein splice form I	Chr20: 9109882 - 9109882	1	Y			Y

ENSRNOG00000018791	Dnlz	DNL-type zinc finger	Chr3: 9159322 - 9159322	1		Y		Y
ENSRNOG00000050780	Fpgs	folylpolyglutamate synthase	Chr3: 17068592 - 17068592	1			Y	Y
ENSRNOG00000033119	Plcb4	phospholipase C, beta 4	Chr3: 135089287 - 135089287	1	Y			Y
ENSRNOG00000006120	Shh	sonic hedgehog	Chr4: 720462 - 720462	1		Y		Y
ENSRNOG00000005306	Lmbr1	limb development membrane protein 1	Chr4: 2330237 - 2330237	1			Y	Y
ENSRNOG00000008939	Nxph1	neurexophilin 1	Chr4: 34824935 - 34824935	1			Y	
ENSRNOG00000008678	Antxr1	anthrax toxin receptor 1	Chr4: 183699441 - 183699441	1		Y		Y
ENSRNOG00000014562	Hnrnpf	heterogeneous nuclear ribonucleoprotein F	Chr4: 215899372 - 215899372	1	Y			Y
ENSRNOG00000017528	Gpr157	G protein-coupled receptor 157	Chr5: 170750766 - 170750766	1		Y		Y
ENSRNOG00000019979,ENSRNOG00000019981	B3galt6,Sdf4	stromal cell derived factor 4,UDP-Gal: betaGal beta 1,3-galactosyltransferase, polypeptide 6	Chr5: 176901531 - 176901531	1	Y			Y
ENSRNOG00000024544	Six3	SIX homeobox 3	Chr6: 8812550 - 8812550	1		Y		Y
ENSRNOG00000020501	Tjp3	tight junction protein 3	Chr7: 11481395 - 11481395	1			Y	
ENSRNOG00000009028	Rnf126	ring finger protein 126	Chr7: 12991216 - 12991216	1			Y	Y
ENSRNOG00000006841	Ano4	anoctamin 4	Chr7: 30094353 - 30094353	1			Y	
ENSRNOG00000011136	Osr2	odd-skipped related transciption factor 2	Chr7: 74217757 - 74217757	1			Y	
ENSRNOG00000042022	H1f0	H1 histone family, member 0	Chr7: 120252050 - 120252050	1	Y			Y
ENSRNOG00000008924	Arhgef12	Rho guanine nucleotide exchange factor (GEF) 12	Chr8: 45864531 - 45864531	1	Y			Y
ENSRNOG00000011071	Nt5e	5' nucleotidase, ecto	Chr8: 95464670 - 95464670	1		Y		Y
ENSRNOG00000014644	Zic1	Zic family member 1	Chr8: 98230074 - 98230074	1	Y			Y
ENSRNOG00000012480	Pxylp1	2-phosphoxylose phosphatase 1	Chr8: 104237724 - 104237724	1	Y			Y
ENSRNOG00000000297	Aqp2	aquaporin 2 (collecting duct)	Chrx: 115747004 - 115747004	1		Y		
